# FFK: Fourier-Transform Fuzzy-c-means Kalman-Filter Based RSSI Filtering Mechanism for Indoor Positioning

**DOI:** 10.3390/s23198274

**Published:** 2023-10-06

**Authors:** Chinyang Henry Tseng, Woei-Jiunn Tsaur

**Affiliations:** 1 Department of Computer Science and Information Engineering, National Taipei University, New Taipei City 23741, Taiwan; tsengcyt@gm.ntpu.edu.tw; 2 Computer Center, National Taipei University, New Taipei City 23741, Taiwan

**Keywords:** indoor positioning, received signal strength indication, Fourier transform, fuzzy c-means, Kalman filter

## Abstract

As indoor positioning has been widely utilized for many applications of the Internet of Things, the Received Signal Strength Indication (RSSI) fingerprint has become a common approach to distance estimation because of its simple and economical design. The combination of a Gaussian filter and a Kalman filter is a common way of establishing an RSSI fingerprint. However, the distributions of RSSI values can be arbitrary distributions instead of Gaussian distributions. Thus, we propose a Fouriertransform Fuzzyc-means Kalmanfilter (FFK) based RSSI filtering mechanism to establish a stable RSSI fingerprint value for distance estimation in indoor positioning. FFK is the first RSSI filtering mechanism adopting the Fourier transform to abstract stable RSSI values from the low-frequency domain. Fuzzy C-Means (FCM) can identify the major Line of Sight (LOS) cluster by its fuzzy membership design in the arbitrary RSSI distributions, and thus FCM becomes a better choice than the Gaussian filter for capturing LOS RSSI values. The Kalman filter summarizes the fluctuating LOS RSSI values as the stable latest RSSI value for the distance estimation. Experiment results from a realistic environment show that FFK achieves better distance estimation accuracy than the Gaussian filter, the Kalman filter, and their combination, which are used by the related works.

## 1. Introduction

As Internet of Things (IoT) technologies grow rapidly, indoor positioning has become one of the crucial emerging IoT technologies [1]. Indoor positioning is utilized by many IoT applications, such as building emergency management [2], smart energy management [3], smart air conditioning controls [4], point-of-interest identification, and occupancy prediction [5,6]. 

Time of Arrival can integrate with Kalman filter and Gaussian mixture models to remove Non-Line of Sight (NLOS) signals for accurate indoor positioning [7], and this can be used for 3D indoor positioning [8]. Time Difference of Arrival [9] and Direction of Arrival [10] can provide enhanced positioning techniques, and they usually require customized sensor configurations or deployments to fulfill their design requirements. 

The Received Signal Strength Indicator (RSSI) fingerprint approach collects stable RSSI values in the fingerprint positions and directly utilizes the stable RSSI values to estimate the distances. Because this direct and economic design provides lower construction costs and simple implementations, RSSI fingerprint is a widely used indoor positioning approach [11,12,13,14,15]. Bluetooth Low Energy (BLE) can provide sensitive RSSI signals to indicate the distance differences for fingerprint establishment [2,14] with low energy consumption, so the combination of BLE and RSSI fingerprinting is an economical and effective method for IoT to deploy indoor positioning applications. 

To establish a stable RSSI fingerprint, the Kalman filter (KF), an effective signal estimation approach, usually integrates with Gaussian-based approaches to filter interfering signals and summarize a stable current RSSI value for the fingerprint, to estimate the distance accurately during the indoor positioning. The Gaussian mixture can identify NLOS signals to assist KF in estimating the latest signals [16,17]. The Gaussian process can identify Gaussian distribution signals to assist KF in improving the signal estimation [18,19]. Gaussian distribution is a popular approach to identifying noise signals to estimate the latest signal, together with KF [20,21,22,23,24,25]. 

KF is a popular latest signal estimation approach for indoor positioning [26,27,28,29]. The Gaussian process is also adopted for RSSI-based indoor positioning [30]. The combination of the Gaussian filter (GF) and KF is a famous integrated approach for indoor positioning based on RSSI. GF can filter NLOS RSSI values to assist KF in estimating accurate RSSI values for indoor positioning [31,32,33]. However, the RSSI distribution may not be a Gaussian distribution, so Fuzzy C-Means (FCM) is adopted to identify RSSI values of major Light of Sight (LOS) signals [34]. Thus, FCM can assist KF in collecting LOS signals without assuming the distribution of RSSI values is Gaussian. In addition, Fourier Transform (FT) is widely used for signal frequency analysis [35,36], so FT can also filter long-term signals in the low-frequency domain.

To estimate stable LOS RSSI values for indoor positioning, we propose a Fourier-transform fuzzy c-means Kalman filter (FFK) based RSSI filtering mechanism. FT converts the overserved RSSI value sequences into the frequency domain. Then, FFK adopts K-means to identify low-frequency values that present stable long-term signals. FCM identifies the major group of LOS RSSI values. The RSSI values in the rest of the small NLOS groups have low membership values for the LOS group, so they belong to NLOS signals. Based on FT and FCM, stable LOS RSSI sequence values are filtered, and KF summarizes the sequence values as the latest LOS RSSI value for distance estimation. Compared with the combination of GF and KF, FFK can estimate the latest LOS RSSI value with better accuracy, because FFK does not treat the distribution of RSSI values as Gaussian, which may not be realistic.

The contributions of the proposed FFK-based RSSI filtering mechanism are summarized as follows:

1. The proposed FFK-based RSSI filtering mechanism is the first RSSI fingerprint method adopting Fourier transform to abstract the long frequency RSSI values for generating stable RSSI fingerprint values to the given distance.
2. FFK adapts FCM to capture LOS RSSI values instead of GF, because the distributions of RSSI values are not Gaussian distributions in reality. Additionally, FCM indicates the cluster degrees of RSSI values to ensure they belong to the LOS cluster. Thus, FCM is a better choice to capture LOS RSSI values than GF.
3. FFK adapts the Kalman filter to summarize the fluctuating LOS RSSI values as the stable latest RSSI value for the distance estimation. As time passes, KF keeps updating the RSSI value for the fingerprint.
4. By combining the Fourier transform, FCM, and Kalman filter, the proposed RSSI filtering mechanism generates the stable current RSSI value for the fingerprint to estimate the distance accurately during indoor positioning.

The rest of this paper is organized as follows. The related works are reviewed in Section 2. Section 3 shows FFK’s detailed design. Section 4 shows FFK’s effectiveness through realistic experiments. Section 5 concludes the work.

## 2. Related Works

Indoor positioning is utilized by many IoT applications. For example, BLE is used to establish an RSSI fingerprint for occupancy estimation for indoor emergency management [2]. IoT-based occupancy-driven plug load management system is implemented as non-intrusive intelligent indoor positioning [3]. The heating, ventilation, and air conditioning system is implemented by indoor positioning with the existing wireless infrastructure [4]. The point of interest data service is deliverable by indoor positioning techniques as its fundamental service [5]. Indoor positioning also delivers occupancy prediction for occupancy-related data services [6]. Thus, indoor positioning is an essential service for many IoT applications. 

Time of Arrival is an effective indoor positioning approach, and it adopts Kalman filter and Gaussian mixture to remove NLOS noise to enhance the accuracy of Time of Arrival [7]. Time Difference of Arrival can enhance the accuracy of Time of Arrival by employing a data-selective approach based on its proposed closed-form least-squares solution that disregards bad measurements [9]. Time of Arrival and Time Difference of Arrival are ideal approaches for 3D indoor positioning based on their design advantages [8]. Direction of Arrival utilizes the azimuth to establish an eigenspace for removing multipath propagation interferences [10]. These approaches usually require customized sensor configurations or deployments to fulfill their deployment requirements. 

The RSSI fingerprint approach directly utilizes the stable RSSI values in the fingerprint positions to estimate the distances. This design enables lower construction costs and simple implementations, and thus the RSSI fingerprint approach is widely used. For example, the maximum RSSI observation was proposed to improve RSSI measurement [11]. An empirical model of indoor RSSI radio map reconstruction was proposed to improve the RSSI accuracy [12]. Adaptive RSSI spring relaxation was proposed with a device-free localization technique for indoor positioning and tracking [13]. Sadowski and Spachos compared WiFi, BLE, Zigbee, and LoRaWAN for use in an indoor localization system, and showed that BLE can provide accurate results with the lowest amount of power [14]. Cho integrated the interacting multiple models and measurement error observer for filtering RSSI data received from mobile nodes [15]. In summary, these works intend to provide stable and accurate RSSI values for indoor positioning. 

Gaussian distribution presents a smooth normal distribution to obtain soothed signal values. Gaussian mixture can identify NLOS signals to assist KF in estimating the latest signals, such as tracking multiple targets in underwater noisy environments [16] and removing noises in the NLOS environment [17]. While the Gaussian process converts the data sequence to Gaussian distribution, the smoothed sequence is good for KF to predict the next latest value. The combination of the Gaussian process and KF can improve attitude estimation with the magnetic field maps [18] and enhance simultaneous localization and mapping with the gradients of the learned magnetic field map [19]. 

To filter the signal noise, Gaussian distribution can identify the noise signals that do not fit the Gaussian distribution. KF can adopt Gaussian distribution to filter the noise to improve the prediction accuracy for different kinds of signal predictions. For example, the combination of KF can adopt Gaussian distribution was adopted for proposing the event trigger algorithm [20], the low-precision numerical representation for efficient Gaussian estimation of high-dimensional problems [21], the adaptive tracking technique for digital global navigation satellite system [22], the resilient state estimation under sensor attacks [23], linear quadratic Gaussian control strategy for concrete caisson deployment for marine structures [24], and the localization method for continuous-wave radar carrier [25]. Thus, KF can utilize Gaussian distribution to achieve accurate latest signal prediction.

KF is a famous latest signal estimation approach for positioning services, such as precise point positioning for global navigation satellite systems [26], the biased localization algorithm based on KF [27], the particle filter combined with KF [28], and RSSI signals from RFID [29]. The Gaussian process can be used for RSSI fingerprint [30]. The integration of GF and KF is adopted for BLE-based RSSI fingerprint [31], combining Wi-Fi channel state information with RSSI [32], and robot indoor location estimation [33].

In reality, the set of LOS RSSI values can be an arbitrary distribution instead of a Gaussian distribution. The usage of Gaussian distribution is to filter out NLOS RSSI values, and clustering algorithms can be sufficient. K-means [37] can capture the major LOS signals as the major cluster, and the minor clusters are NLOS signals. FCM can generate membership value to illustrate the degree of the target value belonging to the major cluster. Therefore, FCM can reveal the degree of RSSI value belonging to the LOS cluster [34]. Thus, FCM can be a better choice than K-means and GF for KF to identify the LOS signals. Also, FT can calculate the frequency of data values [35,36], so FT can identify the long-term stable signal to retrieve stable LOS signals. BLE is supported by most mobile devices, and BLE utilizes low-energy signals which are very sensitive to distance differences [2,14]. Additionally, BLE chips present unique location signatures based on their sensitive signals and unique occupancy identity [38], and thus BLE is ideal for RSSI fingerprint establishment as the effective and economic solution for indoor positioning.

## 3. Fourier-Transform Fuzzy C-Means Kalman Filter Based RSSI Filtering Mechanism

**Figure 1:**
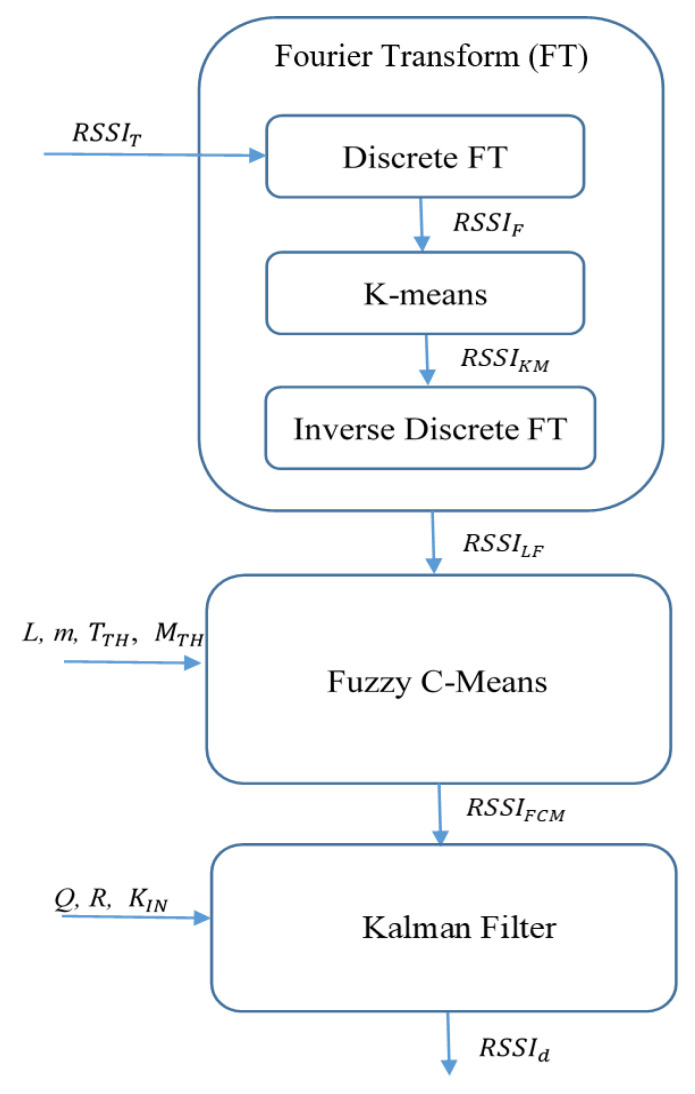
FFK design.

Figure 1 shows the FFK design with the three core components: FT, FCM, and KF. First, FT receives the collected RSSI set, $RSSI_{T}$, from the Bluetooth module, and the RSSI set is treated as an RSSI sequence in the time domain. To obtain the low-frequency values from the set, FT calculates the RSSI values based on the frequency domain. First, the operation of discrete Fourier transform converts $RSSI_{T}\;$ into complex sequences, $RSSI_{F}$, as the data fragments based on the index of the frequency. To dynamically determine the boundary of the low-frequency range, R_LF_, FFK adopts K-means to find the major group of low-frequency values and uses the upper boundary of the group to set $R_{LF}$. Based on $R_{LF}$., $RSSI_{F}$ is filtered and becomes $RSSI_{KM}$. Inverse discrete Fourier transform converts $RSSI_{KM}$ back to the time domain and becomes $RSSI_{LF}$, which represents the low-frequency portions of the original input, $RSSI_{T}$.

Second, FCM divides the input RSSI set, $RSSI_{LF}$, into *L* clusters, and finds the largest major cluster as the LOS set. FCM computes membership degree for each RSSI value of $RSSI_{LF}$ with a weight exponent value, *m*. The membership degree should be higher than the target threshold, $F_{TH}$, to ensure each RSSI value belongs to a suitable cluster. Then, the major cluster is kept as the output, $RSSI_{FCM}$, and other clusters are filtered.

Third, KF treats the RSSI values in a time sequence and breaks the values of $RSSI_{FCM}$ into time intervals. For time each interval having $K_{IN}$ values, the RSSI value is the average value within the interval, and this RSSI value becomes the sample RSSI value for this interval. KF predicts the next RSSI value based on the current RSSI sample value. Then, KF moves to the next time interval and uses the new sample value to calculate the prediction value again. Usually, the system error, *Q*, is set to 0.1 and the measurement error, *R*, is set to 0.01 [35]. FFK does not use the fixed values but uses the initial 100 RSSI values, which reflect major error offsets, to calculate them, in order to obtain better prediction results. $RSSI_{d}$ is the final trained RSSI value from KF and represents the advantages of FT, FCM, and KF for this particular distance. The following subsections illustrate the calculation details for the three major components and provide a summarized algorithm for the entire filtering process.

### 3.1. Fourier Transform Filtering 

Fourier transform is used to transform input data into components in the frequency domain and remove noise from a frequency perspective. Fourier transform treats the entire data and considers all the data instead of deleting some data directly. For discrete Fourier transform of the RSSI sequence of length *N*, the algorithm is depicted as follows:

(1) $$RSSI_{F}=\frac{1}{N}\overset{N-1}{\underset{x=0}{\sum }}\;RSSI_{T}\;exp\left(-\frac{i2\pi ux}{N}\right)$$

where $RSSI_{T}$ is a sequence of data in the time domain, $RSSI_{F}$ is a sequence of data in the frequency domain, N is the number of raw data, and i is an imaginary unit. After the Euler formula $\exp\;\left(ix\right)=\cos x+isinx$, the following formula is obtained:

(2) $$RSSI_{F}=\frac{1}{N}\overset{N-1}{\underset{x=0}{\sum }}RSSI_{T}\;\left[\cos\left(\frac{-2\pi ux}{N}\right)+isin\left(\frac{-2\pi ux}{N}\right)\right]$$

FFK substitutes the original real array into the above formula to get a complex array. This way, low-frequency selection can be performed on the spectrum to improve the data. We use K-means to set the selection of the low-frequency range. The first problem that K-means has to solve is to set the number of groups and the values of the center points. FFK chooses the number of key frequencies whose ratio is higher than 50%, and uses these frequencies as the center points for K-means. This way of setting makes the results of clustering adaptive, rather than simply setting a fixed low-frequency portion. These frequencies represent the characteristics of the original data. It is not like setting the center point randomly, which may result in a poor clustering effect due to excessive concentration. If the number of these center points is less than the minimum number of groups, the clustering cannot be performed, and the threshold reduces until the minimum number of groups is found. The K-means algorithm has the following recursive steps:

Step 1: Set the cluster count *k* and the values of the center points.

Step 2: Calculate the distance between all data points and the center point, and divide all data points into *k* clusters according to the distance from each data point.

Step 3: Average the data points in the group and update the center point.

Step 4: Go back to Step 2 until the center point position no longer changes, and it is considered to be clustered.

After clustering, the major cluster containing the data point with the largest amplitude determines the upper frequency of the selected low frequency, R_LF_. Then, the inverse discrete Fourier transform converts the complex value array of the selected low frequency back to the time domain. The formula of the inverse discrete Fourier transform is as follows:

(3) $$RSSI_{LF}=\overset{N-1}{\underset{x=0}{\sum }}RSSI_{KM}\exp\left(\frac{i2\pi ux}{N}\right)$$

### 3.2. Fuzzy C-Means Filtering

FFK adopts the concept of fuzzy to filter out the errors caused by noise, and the FCM algorithm is depicted as follows.

First, $v_{i}$ is defined as the *i*th cluster center point among *L* clusters, $x_{j}$ is the *j*th data point of $RSSI_{LF}$, and $\mu_{ij}$ is the membership degree of $x_{j}$ belonging to $v_{i}$. The sum of the membership of any data point to all center points is 1, as follows:

(4) $$\overset{L}{\underset{i=1}{\sum }}\mu_{ij}=1,\forall j=1,\ldots ,N$$

Step 1: initialize the membership matrix: randomly generate a number between 0 and 1, and conform to the specification of Equation (4).

Step 2: update the center point location:

(5) $$v_{i}=\frac{\sum_{j=1}^{N}\mu_{ij}^{m}x_{j}}{\sum_{j=1}^{N}\mu_{ij}^{m}}$$

Step 3: update the membership matrix:

(6) $$\mu_{ij}=(\overset{L}{\underset{k=1}{\sum }}{\left(\frac{d_{ij}}{d_{kj}}\right)}^{\frac{2}{m-1}}{)}^{-1}$$

Step 4: calculate the objective function:

(7) $$T=\overset{L}{\underset{i=1}{\sum }}\overset{N}{\underset{j=1}{\sum }}\mu_{ij}^{m}d_{ij}^{2}$$

where *m* represents the weight exponent of membership degree.$\;d_{ij}=|v_{i}-x_{j}|$ represents the distance between the *i*th cluster center point and the *j*th data point. If the target result of the objective function, T, in Step 4, is below the target threshold, $T_{TH}$, which is set to 0.2—or the change of T is below $T_{TH}$—the clustering process is complete, and the algorithm stops. Otherwise, the FCM algorithm goes back to Step 2.

Then, the major cluster is kept, and other clusters are removed. For each RSSI value in $RSSI_{LF}$, if its membership degree is higher than $M_{TH}$, which is set to 0.5, this RSSI belongs to the major cluster, which is also the set of FCM results, $RSSI_{FCM}$.

### 3.3. Kalman Filter

Kalman filter groups the values of $RSSI_{FCM}$ into time intervals. KF computes the prediction value of *k*th interval based on the sample value of *k-1*th interval using the following two recursive steps:

(a) Prediction state

Prediction of covariance:

(8) $$P\left(k|k-1\right)=P\left(k-1|k-1\right)+Q$$

(b) Updating state

Updating of Kalman gain:

(9) $$Kg\left(k\right)=\frac{P(k|k-1)}{P\left(k|k-1\right)+R}$$

Updating of K moment state:

(10) $$x\left(k|k\right)=x\left(k|k-1\right)+Kg\left(k\right)[z(k)-x(k|k-1)]$$

Updating of covariance:

(11) $$P\left(k|k\right)=\left(1-Kg\left(k\right)\right)P(k|k-1)$$

where $x\left(k-1|k-1\right)$ is the sample value inferred from the *k-1*th interval, and $x\left(k|k-1\right)$ is the prediction value at the *k-1*th interval. *P(k-1|k-1)* is the covariance of $x\left(k-1|k-1\right)$, *P(k|k-1)* is the covariance of *x (k|k-1)*, *Kg* is Kalman gain,$\;RSSI_{FCM}$*(k)* is the sample value at the *k*th interval. *Q* is the process noise covariance, so *Q* is also called the system error. A smaller *Q* means more confidence in the predicted value. *R* is the measurement noise covariance, so *R* is also called the measurement error. A smaller *R* means more confidence in the sample measurement. After completing the Kalman filter, the final $\;x\left(k|k\right)$ is $RSSI_{d}$.

### 3.4. RSSI Filtering

The mechanism proposed in this paper is summarized in this subsection. First, we enter the relevant data and parameters. *RSSI_T_* is the RSSI sequence collected in the time domain. *L* is the number of FCM clusters and is set to 3. The three clusters consist of one LOS cluster, which is the target cluster to be reserved for FFK, and two small NLOS clusters, which are the interference noises to be removed. *m* is the weight of the membership, and m is set to 2 as the default FCM setting for normal FCM usage. *Q* is the system error in Kalman filter, and *R* is the measurement error. It outputs a calculated RSSI value, which can be paired with the distance measured during the experiment to obtain $RSSI_{d}$.
**Algorithm 1**. FFK Based RSSI Filtering1. Input: $RSSI_{T}$, *L = 3*, *m* = 2, *R*, *Q*2. Output: $RSSI_{d}$
3. Begin4.     $RSSI_{F}$ = DFT ($RSSI_{T}$)5.     R_LF_ = K-means ($RSSI_{F}$)6.     $RSSI_{KM}$ ← Keep low-frequency values < R_LF_ in $RSSI_{F}$
7.     $RSSI_{LF}$ = IDFT ($RSSI_{KM}$)8.     $RSSI_{FCM}$ = FCM ($RSSI_{LF}$, L, *m*)9.     $RSSI_{d}$ = Kalman ($RSSI_{FCM}$, *R*, *Q*)10. End

In the fourth line of Algorithm 1, the collected RSSI sequence is converted from the time domain to the frequency domain using Discrete Fourier Transform (DFT). K-means are used to determine the low-frequency range. R_LF_ is the upper frequency of the low-frequency range determined by K-means. The low-frequency data is added to $RSSI_{KM}$. The operation of Inverse Discrete Fourier Transform (IDFT) converts $RSSI_{KM}$ back to the time domain. The ninth line uses FCM to classify the low-frequency improved RSSI sequence. The group with the highest total membership degree is regarded as the LOS data and extracted. Finally, the Kalman filter is used to obtain better RSSI values through continuous prediction updates, and the true distances are combined to obtain $RSSI_{d}$ for the distance estimation. The distance estimation is determined by $RSSI_{d}$ [33]:

(12) $$RSSI_{d}=-\left(10nlgd+A\right)$$

where *n* is the signal propagation constant, *d* is the distance to be estimated, and *A* is the RSSI measured value when the distance is 1 m. 

For the computation complexity, DFT is O (*N_T_* ^2^), and IDFT is O (*N_KM_* log *N_KM_*), where *N_T_* is the size of $RSSI_{T}$, and *N_KM_* is the size of $RSSI_{KM}$ [35,36]. K-means is O (*N_F_*), and FCM is O (*N_LF_*), where *N_F_* is the size of $RSSI_{F}$, and *N_LF_* is the size of $RSSI_{LF}$ [34]. Kalman is O (N_FCM_^2^), where N_FCM_ is the size of $RSSI_{FCM}$ [33]. To compare the complexity, DFT and Kalman are similar, and they are higher than IDFT, FCM, and K-means. The computation complexity of GF is O (N), where N is the sample size, and it is similar to FCM. 

## 4. Experiment Results

The experimental equipment used in this paper is BLE development module from Broadcom, which the module number is BCM920737. Bluetooth development module uses the Eclipse IDE environment provided by Broadcom to compile the C programming language, which can be burned into the Bluetooth chip. This only needs to be powered by a USB cable or a small battery. We use BCM920737 to collect RSSI with basic BLE functionality, so other BLE chips can also collect RSSI with their essential BLE implementations. In this experiment, a laboratory with a size of approximately 8 × 5.5$\;m^{2}$ was selected for distance measurement. The measurement distance is from 1 m to 4 m, and a set of data is collected for each additional 0.5 m; a total of seven groups. The collected samples are downloaded by USB cable to the backend desktop computer equipped with AMD Ryzen 3 1200 CPU and 8 GB RAM, and the processing functions of FFK at the computer are written in C and Python.

As we set a high sample rate, many consecutive repeated RSSI values are collected. As the sample rate is set as five samples per second, we can avoid repeated samples and collect sufficient RSSI samples for long-term observation, so the collection duration is 8 h to collect about 144,000 RSSI values for each distance.

We intend to observe stable RSSI samples from two fingerprint points. Since the signals can be unstable and unpredictable without obstacles in reality, we put two chips on the wall in a fixed position without obstacles between them, and we still observed many noises and interferences in the collected RSSI samples. The experiment of each distance was done sequentially by one receiving device, so no time correlation is required. 

### 4.1. Raw RSSI Observation

**Figure 2:**
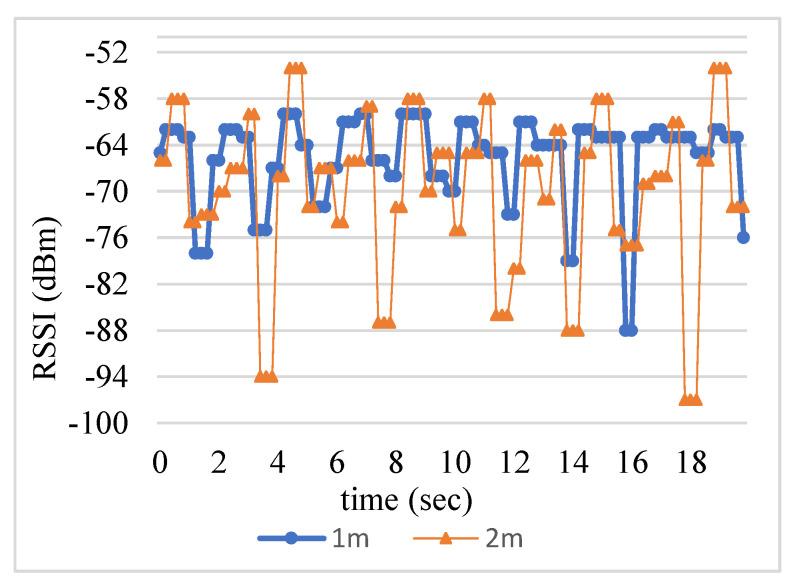
Observed RSSI sets collected at 1 and 2 m.

Figure 2 shows observed RSSI sets collected at 1 and 2 m. The distributions of RSSI sets are arbitrary, and they are obviously not Gaussian distributions. Clearly, they contain noises and require filtering mechanism to remove the noises. Table 1 shows the long distance has the large average observed RSSI. Thus, RSSI can reflect the distance, but filtering the noise from RSSI signals is required. 

**Figure 3:**
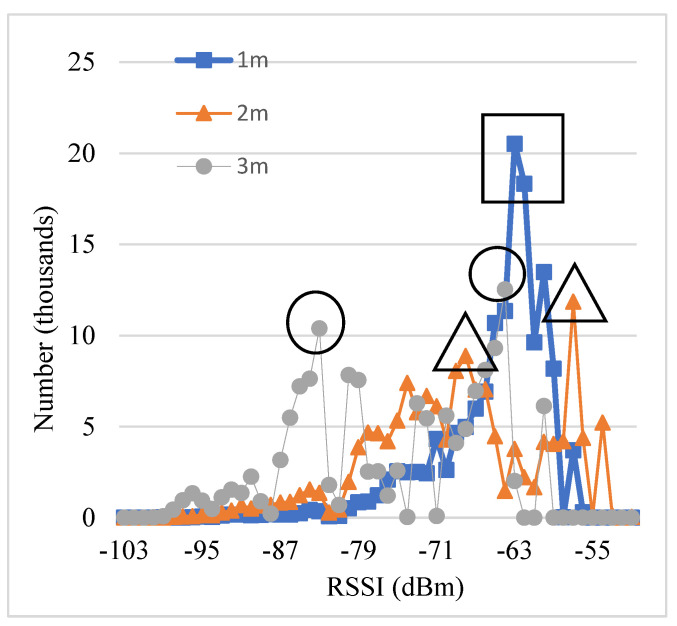
RSSI distributions in 8 h observation. The square, triangle, and circle marks are the peak RSSI values for the distance 1, 2, 3 m.

Figure 3 shows a total of 144,000 RSSI values collected for eight hours at 1, 2, and 3 m, and the number of each RSSI is calculated. In order to facilitate reading, we use different graphics to mark the first two values of the three data. It can be found that the distribution of RSSI is irregular and not concentrated in a small range. RSSI values at different distances have a significant overlap. 

The RSSI set of 1 m is relatively centralized and stable, so the estimation of 1 m is clear based on the peak RSSI values. The peaks of the two meters and three meters are not concentrated. Among these peak RSSI values, one RSSI peak of 2 m at −53 is higher than the two RSSI peaks of 1 m nearby −63. The two peaks of 3 m are at −83 and −64, and one peak of 2 m at −68 is between the two peaks of 3 m. These observations show estimating the distances of 2 and 3 m is more difficult than 1 m because of the irregular overlaps of their RSSI distributions. 

### 4.2. Fourier Transform Filtering

**Figure 4:**
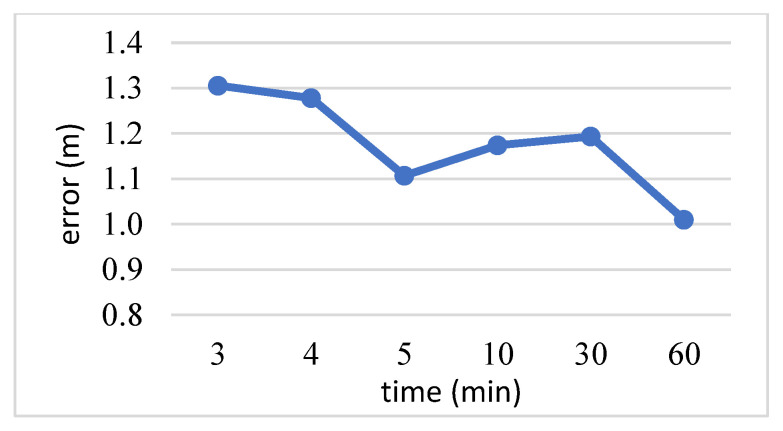
Data collection time for Fourier transform filtering.

Since the test data in practical applications cannot collect data over a long period of time, a short period of test data is substituted into the fitted distance formula to obtain the estimated distance, and then we subtract it from the actual distance to obtain the error distance to verify the mechanism effect. Figure 4 demonstrates that more data helps to improve the accuracy of the mechanism, but testing for an hour reduces the degree of improvement. Therefore, we decided to use 5 min as the length of time in which to collect test data.

**Figure 5:**
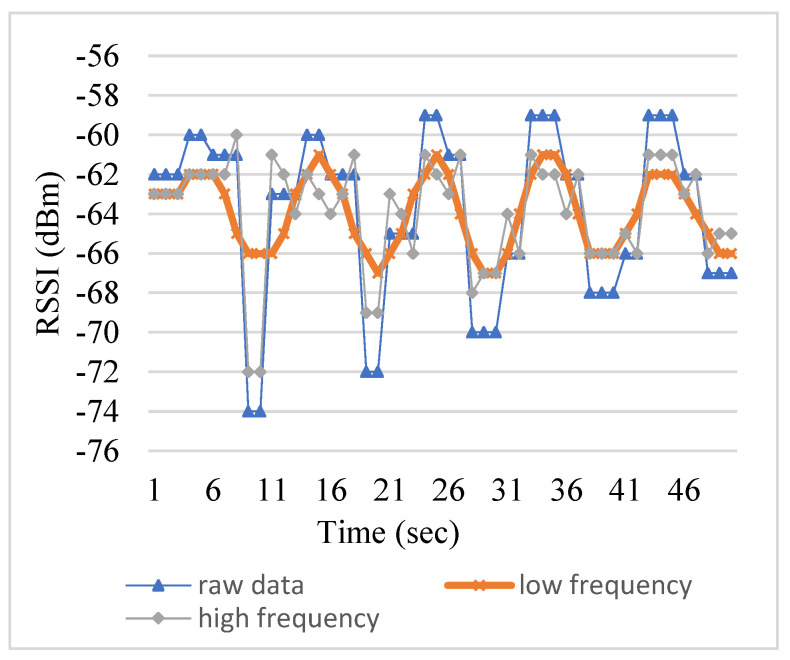
Comparison of high and low frequencies.

Figure 5 shows the comparison of the high frequency and low frequency of the original RSSI in the spectrum. It can clearly be seen that the original RSSI sequence captures the low-frequency portion in the frequency domain and filters out the high-frequency portion that causes the fluctuation. This does achieve a centralized filtering effect.

After comparing the experimental data, it can be found that taking low-frequency data helps reduce the error distance, as shown in Table 2.

In the spectrum, the horizontal axis is frequency. We use the K-means clustering algorithm to determine the range of low frequency. In Table 3, we compare several low frequency ranges, which are maximum amplitude, 20%, 33.3%, and 50% of the lowest frequency. As shown in Table 3, using K-means to adaptively select the low-frequency range has a smaller error distance than a fixed low-frequency range, and is more suitable for various data.

### 4.3. FCM and Kalman Filter

After performing low-frequency filtering on the collected RSSI sequence, we make another improvement on the RSSI sequence. We select the group with the highest total membership degree, and then we filter out the values outside the group. The quarter method divides the input sequence into four equal parts, and takes the middle 50% of the data for calculation. We compare FCM and the quarter method with low-frequency filtering using the Fourier transform (FT) and Kalman filter (KF), as shown in Table 4. The use of FCM has a better effect than the fixed selection ranges of the quarter method.

**Figure 6:**
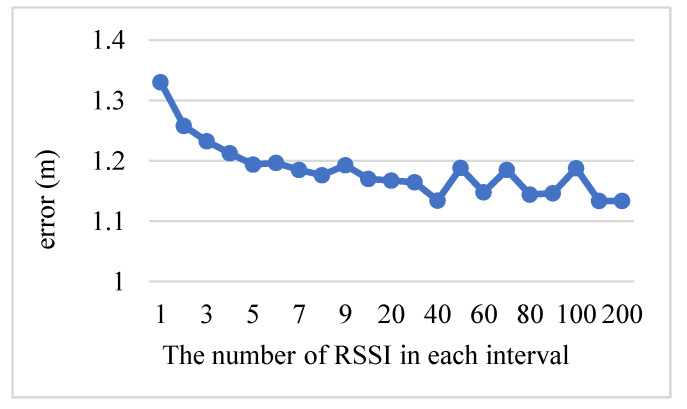
RSSI values of each interval in the Kalman filter.

In the Kalman filter, the input RSSI sequence is divided into several intervals, and the average of the RSSI values within the interval represents the RSSI value for the interval. This approach can effectively reduce errors. Figure 6 demonstrates that the best number in each interval is 40. As this number becomes larger than 40 or smaller than 40, the error is higher. Therefore, each interval in the Kalman filter has 40 RSSI values. 

### 4.4. Comparison of Experimental Results

The procedure of FFK has three steps: (1) FT abstracts low-frequency values, (2) FCM cluster selection, (3) Kalman filter. Then, the test data is verified by the distance conversion formula. Table 5 shows the results of all possible combinations of FFK. The combination of FT, FCM, and KF is the proposed method, and it is the best. Among the other combinations, FCM works the best, KF is the second, FT is the worst. This shows the FCM can successfully capture LOS signals to contribute the best impact. KF can indicate the latest changes as the second contributor. FT can provide the stable signals as the third contributor.

The combination of GF and KF is the compared with related works [23,24,25]. Table 6 shows a comparison of FFK with GF and KF. Because the distribution of RSSI values is arbitrary, instead of a Gaussian distribution, in the realistic experiment, GF may not fit the RSSI distribution. FCM simply identifies the large LOS cluster, so FCM performs better than GF. Due to the arbitrary distribution of RSSI values, the combination of GF and KF is less effective than KF or GF, and KF is better than GF. Even when adding FCM to GF and KF, it becomes worse. In conclusion, FFK works better than the combination of GF and KF, and GF itself. 

## 5. Conclusions

When using RSSI to implement indoor positioning, reducing the errors from multipath effects is an essential issue. Most of the existing methods adopt filtering in the time domain instead of the frequency domain. Since capturing stable RSSI sequences is critical for RSSI filtering, the low frequency of RSSI signals can meet the requirements. Therefore, the proposed RSSI filtering mechanism combines three major methods: Fourier transform, fuzzy c-means, and Kalman filter. First, Fourier transform captures the low frequency in the frequency domain to remove the high-frequency sign portion that causes fluctuations in the RSSI sequence. Then, FCM classifies the low-frequency filtered RSSI sequences. Because most of the collected RSSI values are still LOS data, we choose the group with the highest total membership degree and filter the other groups as noise. Finally, through multiple predictions and updates, Kalman filter calculates the RSSI regularity of each measurement to obtain the latest RSSI value. The experimental results show that the proposed mechanism that combines Fourier transform, FCM, and Kalman filter has the lower error distance compared to the combination of Gaussian filter and Kalman filter. In future works, we will propose a new lightweight RSSI filter running on chips to cooperate with FFK running on a backend computer. FFK provides a stable RSSI for the fingerprints, and the new work will use the stable RSSI as the base to reflect new accurate RSSIs instantly, to update the distance estimation. With the assistance of FFK, the new work will update the new RSSI quickly and accurately, with low computation running on the chip.

## Figures and Tables

**Table 1:** Average of the observed RSSI.

Distance (m)	RSSI (dBm)
1	−66.245
1.5	−68.605
2	−68.535
2.5	−70.895
3	−75.524
3.5	−68.147
4	−83.421

**Table 2:** Comparison of the low-frequency effect.

Data Content	Error (m)
Average of raw data	1.513
Average of low-frequency data	1.409

**Table 3:** Comparison of the low-frequency range.

Range of Low Frequency	Error (m)
K-means	1.107
Maximum amplitude	1.153
20%	1.16
33.3%	1.229
50%	1.199

**Table 4:** FCM and the quarter method.

Method	Error (m)
FT, FCM, KF	1.107
FT, FCM	1.131
FT, quarter, KF	1.3722
FT, quarter	1.427

**Table 5:** Distance errors in different combinations.

Method	Error (m)
FT, FCM, KF	1.107
FT + FCM	1.131
FCM	1.215
KF	1.316
FT + KF	1.335
FT	1.428

**Table 6:** Distance Errors for GF, KF, and FFK.

Method	Error (m)
FFK	1.107
FCM	1.215
KF	1.316
GF	1.414
GF, KF	2.397
GF, FCM, KF	2.914

## Data Availability

The data presented in this study are available on request from the corresponding author. The data are not publicly available due to location privacy.
